# Elevating digital patient education from feasibility to clinical efficacy

**DOI:** 10.1590/1806-9282.20260323

**Published:** 2026-07-20

**Authors:** Ilker Sengul, Demet Sengul, Cagri Akalin

**Affiliations:** 1 Giresun University, Faculty of Medicine, Thyroidology Unit – Giresun, Turkey.; 2 Giresun University, Faculty of Medicine, Division of Endocrine Surgery – Giresun, Turkey.; 3 Giresun University, Faculty of Medicine, Department of General Surgery – Giresun, Turkey.; 4 Giresun University, Faculty of Medicine, Department of Pathology – Giresun, Turkey.; 5 Ordu University, Faculty of Medicine, Department of General Surgery – Ordu, Turkey.

The contemporary landscape of radiation oncology is increasingly defined by the search for scalable, digital solutions to enhance patient comprehension and mitigate treatment-related anxiety. In this context, the dialog initiated regarding the pilot study entitled "Key insights and implementation of a patient-centered education video for managing acute radiation dermatitis in breast cancer: a single-center pilot study," by Hsu and Lin^1^, offers a vital opportunity to reflect on the methodological trajectory required to integrate these tools into gold-standard clinical practice. They considered reply, entitled "In Reply to Sengul and Sengul,"^2^ to our commentary entitled "In Regard to Hsu et al."^3^ concerning their pilot study. Their candor in acknowledging the inherent limitations of their preliminary work is commendable, as is their stated objective of exploring the practical feasibility of integrating a multimedia educational tool into the clinical workflow in mastology^4-9^. The value of such initial investigations, which serve as a necessary precursor to more comprehensive research, is readily apparent. Notwithstanding the evident merits of a feasibility-focused approach, their response invites a collegial discussion on several points of academic and clinical interest. While we commend the authors for their transparency regarding the limitations of their preliminary work, their findings underscore a broader challenge in the field: the "feasibility trap," where the successful implementation of a tool is conflated with its clinical superiority over existing modalities.

## THE IMPERATIVE OF COMPARATIVE UTILITY

The cornerstone of evidence-based medicine rests not merely on the ability to deploy a new intervention without disrupting clinical workflow, but on the rigorous demonstration of its incremental value. The observation that a multimedia tool was integrated without increasing nursing burden is a necessary first step; however, it does not address whether this modality is more effective than enhanced verbal counseling or traditional written materials. In an era of finite healthcare resources, the adoption of digital interventions must be justified by quantifiable improvements in patient-reported outcomes or clinical efficiency. Therefore, the transition from pilot studies to randomized controlled trials is not merely a matter of preference but a scientific necessity to isolate the intervention's efficacy from the "Hawthorne effect" and the baseline standard of care.

## TEMPORAL ALIGNMENT AND SYMPTOM-CONTINGENT INTERVENTION

Perhaps the most salient finding in the current discourse is the temporal mismatch between the delivery of the educational intervention and the manifestation of peak patient distress. Data indicate that patient-reported discomfort reached its zenith during the post-treatment follow-up phase—a period significantly removed from the initial viewing of the educational material. This highlights a crucial pedagogical gap. To maximize the impact of patient education, the "dosage" and "timing" of information must be synchronized with the patient's clinical trajectory. A symptom-contingent or "just-in-time" educational model, whereby patients are encouraged to revisit specific modules as treatment-related toxicity emerges, would likely yield higher therapeutic dividends than a single, front-loaded intervention.

## BRIDGING THE GAP TO HIGH-IMPACT PRACTICE

The journey from Amicus Plato to Magis Amica Veritas requires us to subject our most promising innovations to the highest levels of scrutiny. For digital tools in breast cancer care to be accepted by high-impact journals and clinical guidelines, they must move beyond demonstrating what is possible to proving what is optimal. We must demand a progression in research design that prioritizes longitudinal efficacy, cost-effectiveness, and, most importantly, the alignment of educational support with the lived experience of the patient. Only through such methodological rigor can we ensure that the "common good of the community"—Bonum commune communitatis—is served by the digital transformation of oncology.

## Data Availability

The datasets generated and/or analyzed during the current study are available from the corresponding author upon reasonable request.
